# Anatomo-Functional Origins of the Cortical Silent Period: Spotlight on the Basal Ganglia

**DOI:** 10.3390/brainsci11060705

**Published:** 2021-05-27

**Authors:** David Zeugin, Silvio Ionta

**Affiliations:** Sensory-Motor Laboratory (SeMoLa), Jules-Gonin Eye Hospital/Fondation Asile des Aveugles, Department of Ophthalmology, University of Lausanne, 1002 Lausanne, Switzerland

**Keywords:** review, hyperdirect pathway, neurological disorders, cortical inhibition, basal ganglia

## Abstract

The so-called cortical silent period (CSP) refers to the temporary interruption of electromyographic signal from a muscle following a motor-evoked potential (MEP) triggered by transcranial magnetic stimulation (TMS) over the primary motor cortex (M1). The neurophysiological origins of the CSP are debated. Previous evidence suggests that both spinal and cortical mechanisms may account for the duration of the CSP. However, contextual factors such as cortical fatigue, experimental procedures, attentional load, as well as neuropathology can also influence the CSP duration. The present paper summarizes the most relevant evidence on the mechanisms underlying the duration of the CSP, with a particular focus on the central role of the basal ganglia in the “direct” (excitatory), “indirect” (inhibitory), and “hyperdirect” cortico-subcortical pathways to manage cortical motor inhibition. We propose new methods of interpretation of the CSP related, at least partially, to the inhibitory hyperdirect and indirect pathways in the basal ganglia. This view may help to explain the respective shortening and lengthening of the CSP in various neurological disorders. Shedding light on the complexity of the CSP’s origins, the present review aims at constituting a reference for future work in fundamental research, technological development, and clinical settings.

## 1. Introduction

**Figure 1:**
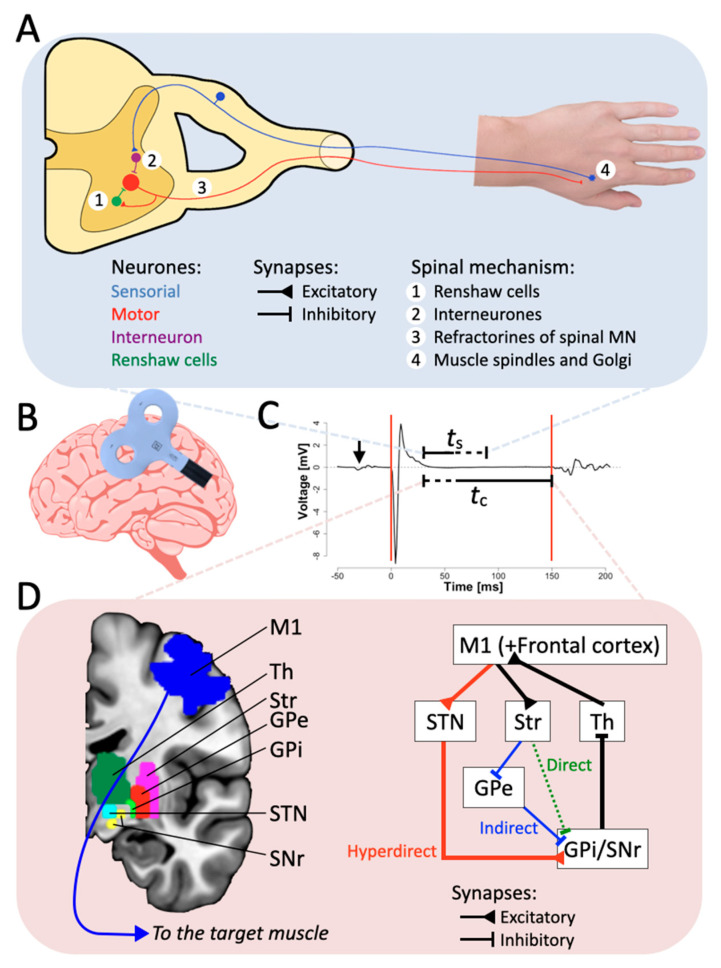
The inhibitory hyperdirect pathway contributes to the CSP duration. (**A**) Schematic representation of the spinal components of the CSP. Excitation of M1 through single TMS pulse reaches spinal interneuronal circuits, which in turn excite hand muscles. The four spinal mechanisms presented in the scheme contribute only to the first 50 ms of the CSP. (**B**) Schematic representation of the TMS coil stimulation in M1. (**C**) Example of an MEP. The red lines represent the usual limit defined for the CSP. “*t*_s_” and “*t*_c_” stand for the duration elicited by the spinal and the cortical part, respectively, and the arrow represents the TMS pulse artifact. (**D**) Simplified schematic diagram of the CBGTC loop, at least partially accounting for the later part of the CSP. Abbreviations: Str: striatum; STN: subthalamic nucleus; GPe: external segment of the globus pallidus; GPi: internal segment of the globus pallidus; SNr: substantia nigra pars reticulata; Th: thalamus.

Performing a movement involves more than just trains of muscular activations. A complex and finely tuned interplay between muscle excitation and inhibition is essential even for very simple movements. One of the most direct marks of motor inhibition, typically measured through transcranial magnetic stimulation (TMS), is the cortical silent period (CSP) [1,2]. The CSP is measured through electromyographic signal recording (EMG) on a target muscle and refers to the period of EMG silence following the elicitation of a motor-evoked potential (MEP) through a single TMS pulse delivered over the contralateral primary motor cortex (M1). Using electrical stimulation, pioneering animal electrophysiology documented the existence of CSP, indicating that excitability of cortical neurons can be reduced after brief and strong stimulation [3,4] and that inhibition lasts for 150–300 ms [5,6]. Similar results have been obtained in human subjects, showing that electrical stimulation of the cerebral cortex through the scalp can elicit CSPs [7], as well as transient functional deficits [8,9]. In parallel with improved comfort for participants [10], the introduction of TMS instead of electrical stimulation to study the characteristics of the CSP allowed the implementation of non-invasive, painless, and more spatiotemporally precise experimental protocols. For instance, one of the most established protocols in the study of cortico-spinal excitability is based on the measurement of single CSPs following a well-controlled MEP elicited by a supra-threshold single-pulse TMS delivered on M1. For both electrical stimulation and TMS, the effects of cortical stimulation on the CSP can be assessed through EMG (see Figure 1B). Nevertheless, despite the largely established reproducibility of the CSP as a direct effect of TMS-induced MEPs and its commonly accepted reliability as a measure of neural inhibition, the neurophysiological origins of the CSP are still under debate. Revealing such origins may boost the understanding of the alterations of the CSP as often observed in several neurological diseases [11,12], possibly linked to physiological or anatomical dysfunctions at the level of the basal ganglia.

The present paper summarizes the relevant evidence for the different possible neurophysiological origins of CSPs. Detailed descriptions of the role of the motor network and cerebellum in CSP dynamics have been provided elsewhere [13,14,15,16,17] and fall out of the present paper’s scope. Here we reconsider the spinal and cortical compounds of CSPs with reference to subcortical activity and cortico-subcortical exchanges, proposing a specific focus on the anatomo-physiological relationship between the CSP and basal ganglia.

## 2. Possible Origins of the CSP

### 2.1. The Spinal Origin

Historically, four non-mutually exclusive and parallel main effects have been considered to account for the spinal components of the CSP [18]. First, TMS over M1 activates a considerable number of spinal motor neurons. This results in the activation of Renshaw cells, inhibiting motor neurons for a maximum of 200 ms [19,20,21]. Second, the activation of inhibitory interneurons after the stimulation of M1 likely inhibits spinal motor neurons for a maximum of <100 ms [22]. Third, despite having a minor influence on the CSP, the refractoriness of the spinal motor neurons is likely the mechanism through which peripheral stimulation contributes to the CSP duration with magnitudes of about 10 ms of inhibition [23]. Fourth, possible effects of brief muscle activation due to TMS delivery on the target muscle’s spindles and Golgi organ might lead to lower excitability of the motor neurons. However, this effect seems negligible compared to the first and second [22].

In the first investigations of the silent period following a punctual stimulation of the spinal cord, the so-called “Hoffmann reflex” protocol has been used to (electrically) stimulate spinal motor neurons and therefore assess spinal excitability [24]. Using a spinal stimulation for a direct nerve activation through the alpha motor neurons, the resulting peripheral silent period is about 50 ms [18,25,26]. Conversely, by means of cortical stimulation (e.g., a single TMS pulse delivered on M1), the resulting silent period is about 150–300 ms long and can last up to 1000 ms [27]. This longer duration with respect to the silent period following spinal electrical stimulation suggests that the CSP comes from the combination of both spinal and cortical components. On this basis, the silent period has been studied for its cortical components in both typical and pathological conditions and has been commonly defined as the “cortical silent period” [28]. Within the first 50 ms of the CSP, it has been observed that the supposedly silent EMG signal in fact presents small variations of EMG activity [20,29,30], defined as “EMG breakthrough activity” [20]. Such breakthrough activity is considered the result of a spinal reflex associated with a sudden activation of the muscle spindles and the following response of the alpha motor neurons in the spine [29]. In this vein, the analysis of EMG breakthrough activity has been considered an important tool to evaluate the status of the spinal cord in both neurological health and disease [30,31], as well as a sign of the presence of both cortical and spinal components in CSP. Furthermore, despite previous claims that the CSP is exclusively of cortical origin [32,33], a recent study by Yacyshyn et al. [34] proposed that the role of spinal inhibitory effects on the CSP has been previously understated. These authors used transmastoid stimulation in order to produce cervicomedullary MEPs, observing that the amplitude of such MEPs did not fully recover even when the stimulation was delivered at an interstimulus interval as long as 150 ms. On this basis, it can be concluded that cervicomedullary MEPs, contrary to the Hoffmann reflex that tests peripheral nerve stimulation, do not impact proprioceptive inflow, which contributes to the cortical component of the CSP. However, these results have to be interpreted carefully, as cervicomedullary MEP amplitude is not directly related to the CSP and does not directly reflect the absence of muscular activity, despite voluntary contraction.

In sum, it seems that different inhibitory phenomena occurring at the level of the spinal cord are responsible for the early part of the CSP, while the later part of the CSP would depend more strongly on cortical components.

### 2.2. The Cortical Origin

As discussed, despite the influence of spinal mechanisms on the CSP duration, converging evidence indicates that the CSP has mainly cortical compounds [32,35]. For example, Fuhr et al. [26] showed that patients presenting extreme sensory neuropathy (with preserved cortical dynamics) do not present shortened CSP, due to intact cortical inhibition mechanisms. Similarly, Chen et al. [35] found, while using paired-pulse TMS stimulation, that indirect waves of corticospinal volleys are reduced at interstimulus intervals between 100 and 200 ms, unlike interstimulus intervals of 50 ms, showing that cortical inhibition is affect by paired-pulse TMS with long interstimulus intervals, out of the range of the first 50 ms of the CSP, induced by spinal mechanisms.

By establishing the independence from excitation and inhibition of M1, Hallett [36] concluded that the CSP and MEP are the results of two independent mechanisms. The CSP has been commonly considered as a peripheral measure of TMS-induced cortical inhibition in M1 [37,38]. However, not only is the length of the CSP affected by the duration of intracortical inhibition in M1 but also by intercortical inhibition mechanisms resulting from weaker neural modulations in brain regions different from M1, including the dorsal premotor cortex (the inhibition of which is associated with CSP shortening) [39]. In this context, Stinear et al. [40] developed the idea that the main function of the CSP at the cortical level is to optimize movement planning. The high amplitude of an MEP generated by TMS is linked to a high intensity of stimulation and, therefore, to the high excitability of M1. Since movement execution needs fine control of possible interferences, unwanted muscular contractions have to be avoided after the corticospinal activation. Stinear et al. [40] proposed that the main function of the CSP is to optimize such a balance between wanted and unwanted muscle contractions by maximizing the MEP signal-to-noise ratio. In particular, the CSP following the MEP would be a way to guarantee precision of movements, assuring movement control while avoiding premature muscular response [41]. It is well known that the excitability of M1 has a direct effect on MEP amplitude [42,43,44]. In order to compensate for such a high excitability, the role of the cortical compound of the CSP is to rapidly compensate the high excitability of M1 by inhibiting M1, to avoid potential secondary excitation of the corticospinal neurons by reducing the probability of unwanted neural firing. Thus, high MEP amplitude is linked to high excitability of M1, resulting in longer CSP. The effect is reversed when MEP amplitude is lower, as a result of lower excitability in M1. The duration of the CSP, as a mark of cortical inhibition, is reduced because the need for stabilization of the efferent cortico-spinal neurons post-excitation is lower. This mechanism is supported by the observed correlation between the amplitude of the MEP and its related CSP [42,43,44]. However, the enhancement of cortical inhibition reflected by the elongation of the CSP can also be obtained without directly affecting the excitability of the entire M1 [45,46]. This means that the process of controlling the CSP duration can also be influenced by indirect inhibitory afferences passing through M1 and/or excitatory afferents to inhibitory sub-areas inside M1 or other brain regions such as the premotor cortex. Furthermore, a CSP can be obtained after TMS stimulation over M1 in the hemisphere ipsilateral to the targeted muscle [47]. Transcallosal mechanisms of inhibition are at the origin of this phenomenon, and contrary to CSP recorded through contralateral stimulation of M1, the ipsilateral CSP is thought to be due exclusively to cortical mechanisms, as patients with lesions at the level of the corpus collosum present partial or absent ipsilateral CSP [48,49,50].

Altogether, previous evidence suggests that the MEP, defined by the combination of amplitude of the muscular response and the duration of the CSP, results from the interplay between excitatory and inhibitory systems, possibly originating from different cortical circuits [51].

### 2.3. Neurotransmitters

The CSP can last for a period up to 1 s [27]. Such important inhibition has been linked to the gamma-aminobutyric acid (GABA) neurotransmitter that, for example, has inhibitory effects up to 1.2 s when GABA_B_ metabotropic receptors are activated in the thalamus [52,53]. GABA is an inhibitory neurotransmitter in the cerebral cortex [54], and its effects on the CSP also have to be considered in relation to the interplay of several other neurotransmitters [55]. GABA_B,_ a subtype of GABA, has a long-lasting activity, the magnitude of which is comparable to the duration of the CSP [5]. Baclofen is a GABA_B_ receptor agonist drug, and its intrathecal administration results in the elongation of the CSP in conditions that otherwise would pathologically shorten CSP, such as generalized dystonia [56]. It is worth mentioning that Baclofen has no effect on spinal inhibitory circuits [57], modulating GABA receptors at the cortical level and reinforcing the idea that the CSP has an important cortical component. In parallel, Werhahn et al. [58] showed that tiagabine, a GABA reuptake inhibitor, can also elongate the CSP. However, such apparent causality between the activation of GABA receptors and the elongation of the CSP might be not as straightforward as it seems. Indeed, McDonnell et al. [59] reported no effect of Baclofen on the duration of CSP, but they observed the elongation of long-interval intracortical inhibition. They suggested that the long-interval intracortical inhibition accounts for magnitude of inhibition, while CSP reflects more its duration. Moreover, dopamine also seems to have a role in the duration of the CSP. Ziemann et al. [60] reported that dopamine agonists enhance the duration of the CSP without affecting the TMS thresholds to activate M1, supporting that mechanisms external to M1 can influence the duration of the CSP. In the same vein, Bäumer et al. [61] reported that the dopamine precursor levodopa elongates CSP in participants affected by Parkinson’s disease. Finally, more recently Thorstensen et al. [62] showed that paroxetine, a reuptake inhibitor of serotonin is able to shorten the duration of the CSP without affecting the muscular activity per se. Interestingly, it also increases the perception of fatigue reported by the subjects (see below).

### 2.4. Behavior and Cognition

Fatigue is defined as the decrease of the maximal force that a muscle can produce and is due to repeated exercise [63]. Interestingly, repeated voluntary contractions at the maximum strength induce fatigue but do not affect the CSP duration [44]. The following loss of force is due mainly to intrinsic muscular mechanisms [64], but the deriving fatigue also leads to modulation of cortical excitability in addition to modulation of muscular physiology [65,66]. Such effects of fatigue can be minimized by specific extensive training of the tested muscle [67]. The cortical effects of fatigue are thought to elongate the CSP duration, as a result of inhibition in M1. However, such a straightforward association between fatigue and stronger cortical inhibition is debated. Levin et al. and Maruyama et al. [68,69] found that cortical inhibition, determined with short-interval intracortical inhibition and measured with paired-pulse TMS, decreased briefly after fatiguing a specific muscle. These authors concluded that the reduction of inhibition could be a compensation mechanism to face some aspects of the lower cortical excitability due to fatigue. At the same time, using single-pulse TMS, Arias et al., Gruet et al., and Liepert et al. [70,71,72] found a lengthening of the CSP during fatigue. To further investigate such an apparent incoherence, Janet L. Taylor and Gandevia [73], proposed that a distinction has to be made between single- and paired-pulse TMS protocols to test the effects of fatigue on cortical inhibition and therefore CSP. Such differentiation may explain that, using a single-pulse TMS approach, the end of the CSP can be considered the mark of the voluntary excitation overcoming the inhibition in M1. Conversely, paired-pulse TMS protocols test a muscle at rest because the second pulse occurs during the CSP. It is therefore likely that previous evidence based on the use of paired-pulse TMS protocols may have to be regarded with caution, supporting the conclusion based on single-pulse TMS that fatigue elongates CSP. This interpretation is also in line with the notion that a distinction must be made between short-interval intracortical inhibition and CSP duration because these two phenomena account for different aspects of M1 inhibition [74].

In addition to the physiological influences on the CSP from neurotransmitters or fatigue, many other aspects of the experiments used to study CSP can in fact influence the CSP itself, including experimental instructions, attention, luminance, and age. With regard to experimental instructions, Mathis et al. [75] found that different types of instructions regarding the level of muscular contraction to reach were given to the subjects, thus testing the effect of intention on voluntary contraction of the muscle and its subsequent inhibition. They observed that with low TMS intensity, when the instructions implied low muscular activation after the TMS, the duration of the CSP was extended. Conversely, when the instructions implied high muscular activation, the CSP was shortened. These effects were not reproduced at high TMS intensities. Moreover, the absence of effects at high TMS intensity indicates a cortical inhibitory effect due to TMS, independent of voluntary’s preparation of contraction. In the same way, the CSP is elongated as the subject is asked to resist to the contraction elicited by TMS [76]. These results underline the cortical components of the CSP, possibly depending on frontal brain areas.

The role of attention in the duration of the CSP is not clearly defined. Attention seems to shorten CSP when the subject has to strongly focus in order to perform a motor task [77,78] or a precision task [79]. However, attention seems to have no effect on the CSP lengthening elicited by repetitive TMS [80,81]. This controversy suggests that attention has to be related to the specific performed motor task. If a task unrelated to motor control is performed, CSP is apparently shortened [82]. It is also possible to shorten the CSP by making the subjects believe their muscular activity has enhanced with placebo-induced [83] or nocebo-induced changes [84]. Altogether, these findings suggest a strong involvement of the frontal cortex in the determination of the CSP.

With regard to luminosity, the CSP has also been modulated by photic activation. Entezari-Taher et al. observed shortening of the CSP after 50–60 Hz photic stimulation [85]. They propose that this effect is due to the indirect pathway between the visual cortex and M1 via the thalamus, reducing GABAergic neuron activity in thalamus, leading to the shorter CSP. However, Cantello et al. [86] found that motor cortical inhibition is enhanced when a flash of light precedes the TMS pulse eliciting MEPs. The domain of research investigating the effect of the visual cortex on the motor one has to be extended, as vision seems to play an important role in the motor control [87].

Finally, some changes to the duration of the CSP are observed as a function of age. In children, incomplete development of GABAergic inhibitory mechanisms leads to longer CSP. The duration is progressively shortened during adolescence [88]. In older age, a majority of studies report a shortening of CSP, but contradictory results have been obtained [1].

## 3. Neurological Disorders

Beside behavioral and cognitive influences on the CSP, many neurological or psychiatric disorders can lead to alteration of the CSP duration. These disorders can be motor and non-motor-related. In both cases, the surplus or deficits of cortical neurotransmitters, in combination or not with dysfunctions in cortical and subcortical areas, can indirectly inform about the origin of possibly altered CSP.

### 3.1. Motor Neurological Disorders

Motor neurological diseases can have dramatic effects on the CSP [11,12]. As the prototype of motor disorder, Parkinson’s disease has been extensively studied and is known to reduce CSP duration [61,89,90,91,92,93,94]. Parkinson’s disease affects motor control by producing bradykinesia, tremors, rigidity, and postural instability [95], but the associated neural alterations do not necessarily comprise M1. It is widely accepted that lesions at the level of the basal ganglia play a major role in leading to the hypokinetic impairments due to Parkinson’s disease [96,97,98]. Interestingly, one of the most effective treatments to contrast the symptoms of Parkinson’s disease is the administration of the dopamine precursor l-DOPA (levodopa; l-3,4-dihydroxyphenylalanine) [99]. As discussed above, dopamine augments the CSP duration [60,100], and it has been suggested that L-DOPA in fact modulates the duration of the CSP by affecting neural activity in the basal ganglia, with specific effects on the inhibition of GABAergic receptors [89,92].

Mirroring the effects of Parkinson’s disease on the CSP, Huntington’s disease also leads to elongation of the CSP. Huntington’s disease is characterized by motor, psychiatric, and cognitive symptoms. The motor symptoms are mainly rigidity and abnormal posturing [101,102]. The origin of Huntington’s disease is possibly due to excessive thalamocortical facilitation in the basal ganglia [92,103,104]. In this vein, Nardone et al. and Schippling et al. [105,106] made the hypothesis that the enhancement of the CSP duration at advanced stages of Huntington’s disease might result from increased inhibition of GABAergic neurons.

Important insights into the effects of motor neurological disorders can be based on studies performed on dystonia. Dystonia is a neurological disorder where patients produce either repetitive movements or sustained muscle contraction, which can lead to unusual posture [107]. Focal hand dystonia patients have shorter CSP [100,101,102], which can be reversed by intrathecal injections of GABAergic agonists, thus elongating CSPs [56]. In particular, shorter CSPs are observed with stronger dystonic contractions, while this negative correlation is not observed when producing voluntary contractions, suggesting the presence of abnormal inhibition mechanisms probably linked to dysfunctions in the basal ganglia [108]. In the same vein, Siebner et al. [109] found a reduction of cerebral blood flow in the lateral putamen with patients suffering from focal arm dystonia, and a correlation has been found between lesions of the putamen and the caudate nuclei and the onset of dystonia due to focal brain lesions [110].

Among other neurological disorders, stroke seems to affect the CSP duration, despite the well-known issue of the homogeneity of lesions between different patients. On the one hand, CSP has been reported as elongated in patients who have suffered from a stroke [111,112,113]. Conversely, Catano et al. [114] reported shortening of the CSP with high muscle contraction in patients severely impacted by stroke [115]. Thus, it is clear that the correlation between stroke and CSP duration is highly variable [116]. Liepert et al. [117] reported elongation of the CSP after unilateral ischemic stroke but also found reduced intracortical inhibition. Intracortical inhibition reduction is a mark of M1 disinhibition, but the CSP is a mark of intercortical inhibition: The results obtained underline two different mechanisms occurring at the same time and the at least partial independence of the CSP from M1 in stroke patients.

Finally, an important influence on the CSP is played by epilepsy [118]. The lengthening of the CSP associated with an epileptic focus involving M1 [119] and generalized epilepsy [120] would possibly suggest a hyperexcitability of inhibitory circuits [120]. However, the available evidence for such an epilepsy-dependent lengthening of the CSP is controversial. While some studies confirmed that epileptic patients presented longer CSPs than normal [121,122,123,124], other studies reported that epileptic patients had normal or shorter CSPs [125,126,127,128]. As in many clinical studies, this controversy might be due to the heterogeneity of the epilepsies included in the studies, little statistical power due to small sample size, and large variability of the exact location of the epileptic focus [129]. Interestingly, in epileptic patients a longer CSP has been observed in response to the stimulation of the unaffected M1, suggesting that epilepsy-related hyperexcitability could spread from the affected hemisphere to contralateral homologous regions [119]. In sum, while there seems to be a consensus that epilepsy affects CSP, a definitive view on the directionality of this influence (longer or shorter CSP) has to be considered still uncertain, possibly due to methodological issues.

### 3.2. Non-Motor Neurological Disorders

It might be argued that the apparently central role of dysfunctions in the basal ganglia in altering the characteristics of the CSP could be biased by the fact that the basal ganglia themselves are often affected by motor neurological disorders taken into consideration to study CSP. However, on the one hand, such disorders also affect other brain regions, including cortical sensorimotor areas, and on the other hand non-motor neurological disorders associated with altered CSP also seem to determine aberrance in the basal ganglia. Indeed, many non-motor psychiatric diseases can dramatically impact the CSP duration (see the review in Farzan et al. [12]) and are associated with dysfunctions in the basal ganglia, even if they do not necessarily trigger purely motor deficits. For example, patients suffering from unipolar major depression present shorter CSP, which is thought to imply GABA_B_ neurophysiological deficits [79,80,81]. Moreover, it seems that depression post-stroke implies dysfunction of thalamocortical projections. These projections are part of the cortico-basal ganglia-thalamo-cortical (CBGTC) loop (see paragraph below), which is involved in motor functions [11]. Furthermore, obsessive–compulsive disorder patients present shorter CSP [130]. Obsessive–compulsive disorder is linked to the dysfunction of the network between motor areas and the basal ganglia [131], as insufficient inhibition of subcortical structures leads to impaired motor control, labeled as hyperfunctioning [84,85,86].

### 3.3. Overview

Many motor and non-motor neurological diseases affect the CSP duration, possibly due to dysfunction in the basal ganglia. Parkinson’s disease produces shorter CSP, but a dopamine receptor agonist can re-elongate the CSP. Huntington’s disease produces longer CSP, possibly due to abnormal inhibitory mechanisms due to GABAergic interneurons. Dystonia is associated with longer CSP, which can be shortened by GABAergic agonists. There is controversy on the impact of epilepsy on CPS, possibly due to methodological issues of the available evidence. On this basis, it could be argued that the association between altered CSP and dysfunctions in the basal ganglia might derive from the fact that all the considered diseases are motor and, therefore, are bound to dysfunctions in the basal ganglia at different degrees. However, non-motor-related diseases (such as depression or obsessive–compulsive disorder) also affect the duration of the CSP and are still related to the basal ganglia. On this basis we propose that the CSP is a reliable measure of motor- and non-motor-related disorders and is associated with impaired inhibition and malfunctioning at the level of the basal ganglia.

## 4. Neural Mechanisms of CSP

### 4.1. Basal Ganglia Involvement in Motor Control and CSP

The basal ganglia interact with different cortical areas, with M1 as the principal output target [132]. Different circuits exist between cortical areas and basal ganglia, each having specific functions. The signals originating in the cortex arrive to the thalamus, which then returns processed signal to motor areas [11,133]. Traditionally, two major neural pathways have been described, the “direct” and the “indirect” pathways [134], as parts of the CBGTC loop [98]. As presented in a simplified schematic way in Figure 1, the direct and indirect pathways have opposite effects. In the direct pathway, excitatory signals from M1 are sent to a specific sub-area of the striatum. The neurons receiving these signals are inhibitory and send inhibitory projections to the internal part of the globus pallidus and to the substantia nigra pars reticulata. Being inhibited, the neurons in the globus pallidus/substantia nigra do not inhibit the thalamus, which in return activates the cortex. Such a direct pathway mainly leads to the activation of M1. In the indirect pathway, excitatory signals from M1 are sent to other specific sub-areas of the striatum again. The neurons receiving these signals are inhibitory and send inhibitory projections to the external part of the globus pallidus, which is inhibited and then does not activate inhibitory projections to the internal part of the globus pallidus/substantia nigra. Thus, the globus pallidus/substantia nigra is disinhibited and, in turn, can inhibit the thalamus through GABA inhibitory projections [135]. The inhibited thalamus does not activate in return the cortex. Such an indirect pathway mainly leads to inhibition of the motor cortices. In this context, there is no clear evidence that TMS over M1 activates either pathway. The neuronal excitation resulting from the TMS-associated rapid discharge of the magnetic field could potentially activate both pathways, but the result of such activation would not lead to a clear inhibition of M1. Preliminary experiments with monkeys and rats have enlightened the involvement of the subthalamic nucleus excitation on the globus pallidus and with motor control [136,137]. Furthermore, Nambu et al. [138] presented the functional significance of the “hyperdirect” pathway for motor control. Such a hyperdirect pathway bypasses the striatum involved in the direct and indirect pathways but converges in the internal part of the globus pallidus [139]. The hyperdirect pathway has a critical role in the CBGTC loop for the development of mature inhibition [140,141], is non-selective [142], and is associated with successful reactive inhibition [68]. Excitatory projections from motor areas arrive in the subthalamic nucleus, the activation of which leads to excitatory projections to the globus pallidus/substantia nigra. Activated as in the indirect pathway, the globus pallidus/substantia nigra send inhibitory projections to the thalamus, leading to suppression of thalamo-cortical output. In their model, Nambu et al. [138] propose that the hyperdirect pathway is the first circuit activated, before direct and indirect, when a voluntary movement is prepared and might play a role with the indirect pathway for inhibition of irrelevant motor programs. Accordingly, it has also been proposed that the hyperdirect pathway is a way to block rapidly a “Go” process [143,144,145], possibly in coordination with the inhibitory indirect pathway [146].

Based on this evidence, we propose that the cortical compounds of the CSP are due to the propagation of the activation signal from the TMS pulse in M1 to the CBGTC loop. In response to the strong and abrupt activation in M1, the hyperdirect pathway would generate a substantial inhibition in the thalamus. Possibly in coordination with the indirect pathway, the inhibition generated in the thalamus would be responsible for the late aspects of the CSP, at least. Such a role of the hyperdirect pathway might also be limited to high-intensity TMS pulse, as low intensity TMS pulses are more sensible to instruction or voluntary control [75]. Since the hyperdirect pathway is a faster response to motor command in the CBGTC loop with respect to the other two pathways, and the main purpose of the hyperdirect pathway is inhibition, it seems reasonable that it can contribute to the inhibition post TMS-pulse excitation. The duration of the inhibition generated would be coherent with the inhibitory GABAergic neurotransmitter present in globus pallidus/substantia nigra stimulated after the activation of the subthalamic nucleus. Graphical representations of the spinal and cortical physiological contributions to the CSP are depicted in Figure 1.

At the cortico-subcortical level, we took into account the CBGTC loop connecting M1 and basal ganglia only in the same, ipsilateral hemisphere. However, several experiments demonstrate the influence of the contralateral hemisphere on the CSP. First, an elongation of the CSP is observed with subjects without transcallosal projections [147]. The role of the basal ganglia in transcallosal communication for M1 inhibition is not set, as deep brain stimulation of the internal globus pallidus, leading to activation of the corticospinal neurons via the internal capsule, does not lead to motor effects [148]. Further studies have to investigate the interplay between the basal ganglia and transcallosal effects on the CSP in order to understand better the role of the corpus callosum in visuomotor coordination.

### 4.2. Hyperdirect Pathway and Motor Neurological Disorders Influencing CSP

Animal research showed that Parkinson’s disease is linked to a weakening of cortico-subthalamic connections, probably due to lower dopamine activity [149], confirming the importance of the hyperdirect pathway and dopamine distribution in the onset of Parkinson’s disease. It is likely that the beneficial effects of dopamine intake on the CSP (elongation) counterbalancing the deficits due to Parkinson’s disease (shortening CSP) could derive from neural dynamics occurring in the subthalamic nucleus [150]. It has been shown that electrical stimulation of the ventral part of the subthalamic nucleus can stabilize the symptoms of Parkinson’s disease [151], probably stimulating dopamine release, and thus indirectly elongating the CSP. This is even less surprising as the ventral part of the subthalamic nucleus accounts for arm and hand control [152], and as most CSP determination for Parkinson’s disease patients is conducted for the muscle of this region. However, broader generalizations of these findings have to be taken with caution. Even if deep brain stimulation of the subthalamic nucleus is a common treatment against Parkinson’s disease [153,154], subthalamic nucleus stimulation alone does not affect the duration of the CSP and therefore the smoothness of motor execution [61]. This support the idea that the inhibition generating the CSP should originate in cortical areas, projecting then to the subthalamic nucleus. Unlike Parkinson’s disease, for Huntington’s disease the role of the subthalamic nucleus is debated. Schroll et al. [155] do not present results showing that Huntington’s disease is due to the lesion of the subthalamic nucleus or dysfunction in dopamine circuitry. At the same time, a study on mice with hypo-functioning hyperdirect pathway presents hyperkinesic symptoms specific to Huntington’s disease [156]. The neurological dysfunction of Huntington’s disease are often re-evaluated [135], and the hyperdirect pathway might still be relevant in the understanding of the disease and the related effects observed on the CSP. For dystonia, it is worth noting that dystonic symptoms might result from maladaptive neuroplasticity in thalamo-basal circuits [157], which would provide already “distorted” signals to the cortex. This could be reflected in unbalanced exchanges between the hyperdirect and indirect pathways, which may explain the development of dystonic behaviors [158,159,160] in parallel with a hyperfunctional direct pathway [161]. Finally, acute ischemic stroke leading to hemiballism-hemichorea, a movement disorder characterized by possible violent involuntary movement, has been shown to be associated with lesion in the subthalamic nucleus and the hyperdirect pathway [162].

In sum, the TMS pulse over M1 could lead first to activation of the inhibitory hyperdirect pathway in the CBGTC loop. The CSP observed after an MEP seems to be the consequence of the inhibition due to first the hyperdirect pathway and then the inhibitory indirect pathway. This hypothesis is reinforced by evidence showing pathological duration of the CSP in motor neurological disorders associated with the CBGTC loop.

## 5. Conclusions

The present paper puts emphasis on the anatomo-functional origins of the CSP. In our view, the CSP is determined by both spinal and cortico-subcortical mechanisms. The spinal components account for up to the first 150 ms of the CSP. Then, despite spinal inhibition, cortical mechanisms also influence the CSP, as a mark of M1 inhibition, mediated by GABAergic neurons after an important cortical activation eliciting MEPs. The role of this inhibition is to prevent unwanted movements from occurring. Behavioral and cognitive factors can influence the CSP duration, as well as motor and non-motor neurological disorders. Many of these disorders present anomalies in the basal ganglia, suggesting a central role of this region in determining the characteristics of the CSP. Given that (i) motor output is mediated by the CBGTC loop and (ii) the hyperdirect pathway in the CBGTC loop has important interactions with the indirect pathway for motor inhibition, we propose that the later part of the CSP is mainly influenced by the inhibition induced by the interactions of hyperdirect and indirect pathways crossing at the level of the basal ganglia. A better comprehension of a multifaceted origin of the CSP might lead to better understanding of the physiopathology of motor and non-motor neurological disorders.

## Abbreviations

CSP	cortical silent period
TMS	transcranial magnetic stimulation
MEP	motor-evoked potential
M1	primary motor cortex
GABA	gamma-aminobutyric acid
L-DOPA	levodopa
CBGTC	cortico-basal ganglia-thalamo-cortical
